# Development of an Innovative Financial Literacy and Preparedness Program for Family Caregivers

**DOI:** 10.1093/geroni/igaa057.1148

**Published:** 2020-12-16

**Authors:** Katherine Judge, Sam Fazio

**Affiliations:** 1 Cleveland State University, Cleveland, Ohio, United States; 2 Alzheimer’s Association, Chicago, Illinois, United States

## Abstract

Informal caregivers provide the bulk of daily care and assistance to older adults needing help. Tasks range from assisting with transportation, coordinating care and appointments, household tasks, emotional and social support, and personal care. Caregivers also assist with financial care-related issues. This assistance ranges from helping pay bills, making health-care decisions, to paying out-of-pocket care expenses. Research on financial care-related issues greatly lags behind other areas of caregiving. Additionally, few programs have been developed that specifically address these financial issues and how best to provide timely and personalized information for families. The following poster will present an innovative program that addresses these gaps within the literature and fills the void facing families in navigating key financial care-related decisions. Funded by the Administration on Community Living, the evidence-informed program was developed based on findings from a comprehensive literature review, an environmental scan, market analysis, and feedback from focus groups. The program includes educational information, skills-training, and resources for caregivers across the following content areas: Introduction to Costs of Caregiving, Benefits of Early Planning, Avoiding Financial Abuse and Fraud, Conversations about Finances, Assessing Financial Needs, Creating Action Plans, and Finding Financial Support. The program also addresses specific financial needs facing caregivers of individuals with Alzheimer’s disease and related dementias. Discussion will highlight key aspects of the program, including the standardized yet flexible and tailored approach for addressing families’ specific financial care-related needs, along with next steps in program implementation and evaluation.

